# Case of Acute Graft Failure during Suspected Humoral Rejection with Preserved Ejection Fraction, but Severely Reduced Longitudinal Deformation Detected by 2D-Speckle Tracking

**DOI:** 10.1155/2014/173589

**Published:** 2014-06-11

**Authors:** Tor Skibsted Clemmensen, Hans Eiskjær, Pernille B. Kofoed-Nielsen, Søren Høyer, Steen Hvitfeldt Poulsen

**Affiliations:** ^1^Department of Cardiology, Aarhus University Hospital, Skejby, Brendstrupgaardsvej 100, 8200 Aarhus, Denmark; ^2^Department of Clinical Immunology, Aarhus University Hospital, Skejby, Brendstrupgaardsvej 100, 8200 Aarhus, Denmark; ^3^Department of Pathology, Aarhus University Hospital, Skejby, Brendstrupgaardsvej 100, 8200 Aarhus, Denmark

## Abstract

This case displays limited utility of left ventricular ejection fraction to detect acute graft failure due to microvascular vasculopathy and suspected humoral rejection. Despite severe and progressive graft failure, clinically and by right heart catheterizations, left ventricular ejection fraction remained unchanged, indicating need of more reliable noninvasive methods for graft function surveillance. Global longitudinal strain relates to clinical heart failure, filling pressure, and cardiac index during suspected humoral rejection and microvascular dysfunction in this HTX patient. We suggest routine monitoring of graft function by global longitudinal strain as supplement to routine left ventricular ejection fraction and diastolic Doppler measurements.

## 1. Introduction

Heart transplant recipients are at increased risk to develop myocardial dysfunction due to rejections, fibroses, and vasculopathy. Standard surveillance of graft function after heart transplantation (HTX) includes measurement of left ventricular (LV) ejection fraction (EF) and diastolic function by mitral valve Doppler flow. Our clinical experience shows that left LV-EF often presents itself within normal range during acute rejection, despite severe vasculopathy, suggesting that LV-EF is an inappropriate parameter for detecting impaired myocardial function. Global longitudinal strain (GLS) by 2-dimensional speckle tracking (2D-STE) represents a new angle-independent echocardiographic tool for global assessment of LV systolic function. GLS is a direct measurement of myocardial longitudinal deformation, less dependent on heart rate and loading conditions than LV-EF and diastolic Doppler measurements [1]. This case displays limited utility of LV-EF to detect acute graft failure due to microvascular vasculopathy and suspected humoral rejection. On the contrary, GLS related to clinical heart failure, filling pressure, and cardiac index during the clinical course.

## 2. Case Presentation

A 49-year-old female heart-transplanted (HTX) patient was hospitalized on December 3, 2010, due to one week with dyspnea (NYHA IIb) and dizziness. Blood pressure was 143/100, heart rate 125 (sinus rhythm (SR)), and saturation 97%. Electrocardiogram showed 1-2 mm ST-depression in V2–V6. Blood samples revealed myocardial necrosis, TNT 148 ng/L, and CKMB 6 *μ*g/L. CRP was slightly elevated, 20.3 mg/L, and Nt-Pro-BNP was moderately elevated, 2018 ng/L. Chest X-ray was without acute pathology.

The patient was priorly transplanted in March 2008 due to terminal ischemic heart failure. Donor was an 18-year-old male; both donor and patient were blood type A. Transplantation was completed with 4 HLA mismatches (HLA-A30; B7; DRB1*07, *12). No prospective cytotoxic crossmatch was performed. Cold ischemic time was three hours; the heart started spontaneously in SR. Echocardiography showed normal biventricular systolic function. First endomyocardial biopsy (EMB) showed signs of ischemic transport damage, afterwards no treatment demanding rejections. Coronary angiographies showed normal coronary arteries within the first two years after HTX.

**Figure 1:**
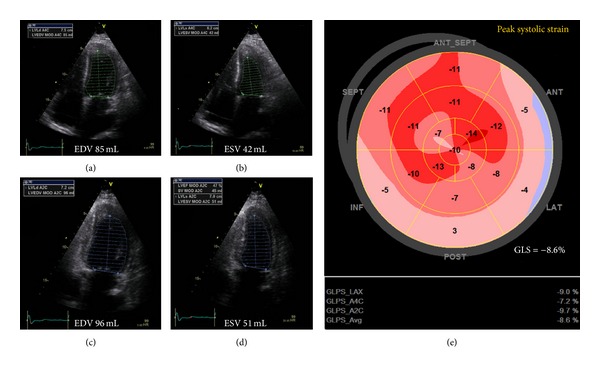
Echocardiography 29.12.2010. (a) and (b) End-diastolic and end-systolic apical 4-camper view. (b) and (c) End-diastolic and end-systolic apical 2-camper view. LV-EF 49%. (e) Bulls plot of global longitudinal strain = −8.6%.

**Figure 2:**
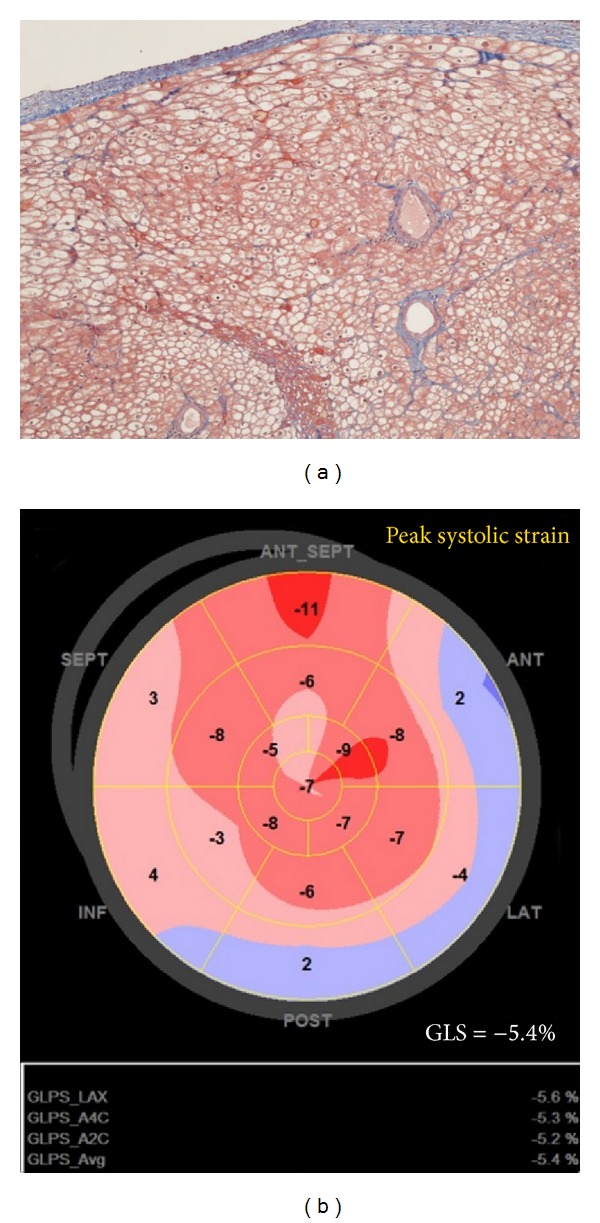
(a) Endomyocardial biopsy, cardiac explant 11.01.2011, right ventricle, and Masson's trichrome ×125, showing myocytolysis and thrombotic occlusion of minor arterial branches. (b) Echocardiography 06.01.2011: bulls plot of global longitudinal strain = −5.4%. LV-EF at this point 49% (LV-EDV 82 mL, LV-ESV 42 mL).

Echocardiography at hospitalization (December 2010) revealed significant restrictive filling with preserved LV-EF but decreased longitudinal biventricular function (EF 55%, E/A-ratio 2.4, E-deceleration time 60 ms, IVRT 46 ms, LV-global longitudinal strain (GLS) −8.9%, TAPSE 1 cm, and right ventricular (RV)-GLS −6.4%). Intravenous methylprednisolone of 1 g daily for three days was immediately initiated, due to severe suspected acute cellular rejection. EMB and coronary angiography on December 6 showed 1R cellular rejection, significant myocytolysis, and angiographically normal coronary arteries. Intravascular ultrasound examination of all three major branches on December 13 confirmed normal coronary arteries, and repeated EMB showed 1R cellular rejection with significant myocytolysis. C4d was only positive in necrotic areas; no significant histological signs of humoral rejection were described. A DDD-pacemaker was implanted due to symptomatic episodes with sinus bradycardia and nodal rhythm. On December 16 a flow cytometric crossmatch performed on donor T- and B-cells was found negative, not supporting the diagnosis of humoral rejection. Luminex analysis showed three donor-specific HLA antibodies, anti-DR12 (5.200 MFI), anti-DR7 (2.500 MFI), and anti-DR52 (3.500 MFI) indicating some donor reactivity. Serological markers of myocardial necrosis remained elevated. The clinical condition declined with decreasing diuresis, increasing s-creatinine, and dyspnea (NYHA IV). Right heart catheterization on December 28 showed severely elevated biventricular filling pressure and heart failure (right atrium (RA) 23/14 mmHg (20), pulmonary artery (PA) 32/20 mmHg (24), pulmonary capillary wedge pressure (PCWP) 27/10 mmHg (18), and cardiac index (CI) 1.7 L/min, SvO2 53% SaO2 92%). At this point Nt-Pro-BNP was increased to 5034 ng/L. Former EMBs were reevaluated revealing occlusion of several minor arterial branches. Overall, humoral rejection was most likely the cause of microvascular dysfunction. On December 29, plasmaferese, intravenous methylprednisolone, immunoglobulin, and rituximab were initiated. Echocardiography showed unchanged preserved LV-EF and severely reduced biventricular longitudinal function (LV-EF 49%, LV-GLS: −8.6%, TAPSE: 0.8 cm, cm/s, RV-GLS: −6.1%) (Figure 1). A new Luminex analysis on December 30 confirmed the previous findings of three donor-specific antibodies, supporting the diagnosis acute humoral rejection. Right heart catheterization on January 6 showed decreasing CI with severely elevated biventricular filling pressures (RA: 25/14 mmHg (21), PA: 28/15 mmHg (22), PCWP: 22/13 mmHg (17), CI: 1.1 L/min, and SvO2: 36-37%). LV-EF was still near normal, but longitudinal function of both ventricles was further decreasing (LV-EF: 49%, LV-GLS: −5.4%, TAPSE: 0.3 cm, and RV-GLS: −3.4%) (Figure 2). Levosimendan was added to the treatment. On January 7 the patient developed cardiac arrest and was successfully resuscitated and cardiopulmonary support was initiated. The patient was listed for re-HTX as urgent call. Successful re-HTX was performed on January 11.

Autopsy findings of the explanted heart revealed histological signs of humoral rejection with severe graft vasculopathy in the microvascular system by means of occlusive intima fibrosis involving myofibroblast proliferation, macrophages, and lymphocytes. In the myocardium, severe diffuse myocytolysis and bleeding were seen (Figure 2). No signs of infection, significant cellular rejection, or epicardial vasculopathy were seen. Mixed beads analysis from 20.06.2011 was negative with no sign of MIC-A antibodies.

## 3. Discussion

The diagnostics of humoral rejection and microvascular dysfunction are challenging in the clinical setting. Despite relevant examinations, treatment for humoral rejection in this present case was initiated 26 days after hospitalizing. Microvascular dysfunction and humoral rejection should be suspected in HTX patients with clinical heart failure, without evidence of cellular rejection or epicardial vasculopathy. The combination of preserved LV-EF and severely reduced GLS indicates impaired subendocardial perfusion. Longitudinal myocardial function predominantly represents function of the subendocardial longitudinally oriented fibers. These fibers are the most sensitive for impaired myocardial perfusion, edema, and fibrosis [2]. GLS by 2D-speckle tracking is a direct global angle independent measure of myocardial longitudinal deformation, less dependent on heart rate and loading conditions than EF and diastolic Doppler measurements [1]. Previous studies have shown significantly reduced GLS in stabile HTX patients as compared to healthy subjects [3, 4] and an important prognostic marker within the first two years after HTX [5, 6]. However, the correlation between GLS and microvascular dysfunction in HTX patients remains to be determined.

In this case, progressive heart failure was confirmed by elevated filling pressures, decreased CI, and long-axis analysis of both ventricles, whereas LV-EF was only slightly affected and unchanged during the clinical course. When evaluating myocardial function in HTX patients, LV-EF is often within normal range despite significant cardiac rejection or vasculopathy, indicating that LV-EF is unreliable in graft function surveillance.

## 4. Conclusion

In contrast to LV-EF, GLS relates to clinical heart failure, filling pressure, and CI during microvascular dysfunction and suspected humoral rejection in HTX patients. We suggest routine monitoring of graft function by GLS as supplement to routine LV-EF and diastolic Doppler measurements.

## References

[1] Dandel M, Hetzer R (2009). Echocardiographic strain and strain rate imaging—clinical applications. *International Journal of Cardiology*.

[2] Laøgstrup BB, Haøfsten DE, Christophersen TB (2012). Correlation between left ventricular global and regional longitudinal systolic strain and impaired microcirculation in patients with acute myocardial infarction. *Echocardiography*.

[3] Saleh HK, Villarraga HR, Kane GC (2011). Normal left ventricular mechanical function and synchrony values by speckle-tracking echocardiography in the transplanted heart with normal ejection fraction. *The Journal of Heart and Lung Transplantation*.

[4] Syeda B, Höfer P, Pichler P (2011). Two-dimensional speckle-tracking strain echocardiography in long-term heart transplant patients: a study comparing deformation parameters and ejection fraction derived from echocardiography and multislice computed tomography. *European Journal of Echocardiography*.

[5] Sarvari SI, Gjesdal O, Gude E (2012). Early postoperative left ventricular function by echocardiographic strain is a predictor of 1-year mortality in heart transplant recipients. *Journal of the American Society of Echocardiography*.

[6] Eleid MF, Caracciolo G, Cho EJ (2010). Natural history of left ventricular mechanics in transplanted hearts: relationships with clinical variables and genetic expression profiles of allograft rejection. *Journal of the American College of Cardiology Cardiovascular Imaging*.

